# Leave no one behind: Global Analysis on monitoring childhood vaccination timeliness and catch-up using data from the WHO/UNICEF joint reporting form on Immunization

**DOI:** 10.12688/gatesopenres.16398.1

**Published:** 2026-09-01

**Authors:** M. Carolina Danovaro-Holliday, Ignacio Castro-Aguirre, Mitsuki Koh, Inae Carolyn Kim, Yoann Nedelec, Marcela Contreras, Naor Bar-Zeev, Martha Velandia-Gonzalez, Marta Gacic-Dobo

**Affiliations:** 1 World Health Organization, Geneva, GE, 1211, Switzerland; 2 Pan American Health Organization, Washington, DC, 20037, USA

**Keywords:** Immunization, Catch-up, timeliness, delayed vaccination, global, immunization information systems

## Abstract

In the years following the Covid-19 pandemic, immunization programmes have increasingly focused on the capacity to conduct and monitor catch-up vaccination—for doses administered later than the recommended schedule. However, it is unclear whether information systems are able to monitor delayed vaccination. We analyzed WHO/UNICEF Joint Reporting Form data from 194 countries (2016–2022) to understand if systems for tracking delayed childhood vaccination are available and quantify catch-up doses of the first dose of a measles-containing vaccine (MCV1). For Latin American and Caribbean countries, we also explored data for the third dose of diphtheria-tetanus-pertussis vaccine (DTP3).

We assessed the completeness and consistency of reporting, the proportion of delayed doses, and vaccination coverage considering both timely and delayed administration. Analyses were disaggregated by WHO Region, World Bank income classification, and Gavi support eligibility.

Results show substantial variability across regions and income groups in countries’ ability to capture delayed doses. Between 2016 and 2022, the proportion of countries reporting a system to record delayed doses ranged from 55.5% to 64.4% annually, with the Region of the Americas (AMR) consistently reporting a higher proportion (77.1%–82.9%). Among countries with available data, the median proportion of delayed MCV1 doses was 4.4%, suggesting that including catch-up doses in cohort monitoring would increase reported coverage by approximately 4 percentage points. This increase was smaller in Gavi-eligible countries compared with non-eligible countries.

The study summarizes global differences in information systems to monitor catch-up vaccination, presents examples of cohort coverage monitoring and illustrates the need to further improve immunization information systems and explore the potential for improving vaccination coverage and population immunity through catch-up activities.

## Highlights

• Following the Covid-19 pandemic and the associated declines in childhood vaccination coverage, the implementation and monitoring catch-up vaccination has become a global priority. This requires monitoring “delayed doses”, or doses given after the recommended age and reinforce the importance of cohort monitoring.
• Since 2016, data has been collected globally on the ability of information systems to track delayed vaccination including doses of the first measles-containing vaccine (MCV1). For countries of the Americas, data for delayed MCV1 and other vaccines has been collected for over 20 years.
• While in 2022, WHO Member States reported having a system to capture late MCV1 doses increased to 119 (61%), from 106 countries in 2016; only 97 of those 119 who said yes in 2022 had a “consistent” reply over time. Variation by WHO region and by income-level is observed in reporting the presence of a system to capture late doses.
• For 2022, 87 of 186 (47%) WHO Member States that submitted the JRF reported delayed doses ranging from <1% to 53% of MCV1 doses administered. The broad range, and values of 50%, raise concerns about the quality of some of those figures, as such values are unlikely to reflect what immunization programmes do.
• In addition to efforts to ensure timely vaccination, to close immunity gaps it is essential not only to implement catch up vaccination, but also to improve capturing delayed vaccination and monitoring the percentage of doses administered to various age cohorts over time.

## Introduction

Since the inception of the Expanded Program on Immunization (EPI) in 1974, remarkable achievements have been made. Estimates of deaths prevented, suggest that vaccination has averted 154 million deaths, including 146 million in children aged under 5 years. ^1– 4^ We have transitioned from a world where less than 20% of children were vaccinated to achieving and sustaining coverage of around 85% three doses of diphtheria-tetanus-pertussis-containing and polio vaccines (DTP3, Pol3) and 83-86% for a dose of measles-containing vaccines (MCV1) in the last decade. ^5, 6^ Currently, routinely administered vaccines help prevent about 20 life-threatening vaccine-preventable diseases (VPDs) avoiding an estimated 3.5 to 5 million deaths annually. ^7^

In August 2020, the World Health Assembly endorsed the Immunization Agenda 2030 (IA2030), which envisions a world where everyone, everywhere, at every age, fully benefits from vaccines to improve health and well-being. ^8, 9^ The launching of the IA2030 coincided with the Covid-19 pandemic, caused by SARS-CoV-2. ^10^ The pandemic and related control measures significantly affected immunization programs worldwide: immunization coverage declined, vaccination campaigns were disrupted, and the recovery was highly inequitable between and within countries. ^11– 14^

Given the increased immunity gaps resulting from the backsliding in vaccination coverage and campaign disruptions, and the resulting vaccine-preventable disease outbreaks, in 2023, immunization partners launched the “Big Catch-up: An Essential Immunization Recovery Plan” (BCU). The BCU is a global strategy to close gaps in immunization focusing on three aspects: 1) reaching children who missed vaccination since 2020 ( *Catch-up *), 2) restoring routine vaccination coverage to at least 2019 levels ( *Restore*), and 3) strengthening immunization systems ( *Strengthen*). ^15– 17^

In general, catch-up vaccination refers to vaccinating an individual who for whatever reason is “late” to receive a vaccine for which he or she is still eligible. While timely vaccination reduces the time an infant is unprotected, catch-up vaccination is still preferred to no vaccination. ^18, 19^

Most countries rely on aggregated information systems that compile doses administered from tally sheets. However, effective monitoring of delayed or catch-up vaccination requires systems capable of capturing vaccination by specific age groups. This analysis provides the first global overview of the availability of age-disaggregated data for early childhood vaccination and the extent to which delayed vaccination is monitored, based on data reported to WHO and UNICEF. These findings aim to inform efforts to strengthen catch-up vaccination strategies while advancing progress toward higher and more timely coverage.

## Methods

### Data sources

We used data reported to WHO and UNICEF through the Joint Reporting Form for immunization (JRF). The JRF is the only global and the most comprehensive immunization data reporting mechanism. There is a common global set of core questions and specific questions for countries served by the different WHO Regional Offices. For example, a JRF form completed by an African country will be somewhat different to the one filled-in by a country of the Americas or of the Western Pacific Region. More specific information on the JRF and the data collection process can be found elsewhere. ^20^ We used JRF data from 194 WHO Member States for the years 2016 to 2022. We start with the first year these data were collected and stop in 2022 because the questions changed in 2024 (for 2023 data). In addition, for countries of the Americas, we used two variables from the regional JRF from 2006. ^21^

### Variables and definitions used

The JRF variables analyzed in this paper included: 1) number of MCV1 doses administered to children in their first or second year of life depending on the recommended schedule, i.e., “timely doses”; 2) answers to the question “Does your recording system allow for capture of data, at the national level, on the number of delayed or late doses administered?” yes/no and 2) answers to “If MCV1[first dose of a measles-containing vaccine] is recommended in the routine schedule at less than 12 months (i.e. 9-11 months), how many doses of MCV1 were administered at 12-23 months in {Year}?” or “If MCV1 is recommended in the routine schedule at 12 months or later, how many late or delayed MCV1 doses were administered in {Year}”. These JRF questions are asked in all WHO regions.

Data on delayed doses of other vaccines were not collected, except for countries of the Americas. For countries of the Americas, in addition to analyzing MCV1 delayed doses, we also analyzed “timely” and “delayed doses” of the third dose of a diphtheria-tetanus-pertussis vaccine (DTP3).

We defined as “timely” doses those administered in the first year of life when MCV1 is recommended in infancy, and those administered in the second year of life when MCV1 is recommended for children >=12 months. We defined as “delayed doses” those reported when answering the second question above, i.e., given to children >=12 months when MCV1 is recommended in infancy and those given to children >=24 months when MCV1 is recommended in the second year of life.

As there is no single definition of “late” vaccination or consensus on what represents a “delayed” vaccine dose or, using a more positive connotation, a “catch-up dose”, ^22^ the definition of “delayed” dose used here is consistent with the terminology used in global data collection efforts and in the recent catch-up guidance.

### Analysis

We computed completeness of reporting to WHO/UNICEF over the seven years included.

As it is not possible to assess the quality of the self-reported data from Member States, for the question on the information system, as a proxy for data quality, we explored consistency, with “consistent reporting” defined as keeping the same answer over the reporting period or going from “no” to “yes”, with “yes” remaining as the most recent answer. Switching from reporting “yes” to “no” was considered inconsistent reporting. We also quantified instances when a country indicated not having a system to capture delayed MCV1 doses separately from timely doses and then reported a value in the field related to delayed doses. Only since 2020, the JRF includes a logical skip pattern to prevent countries who report not having a system to capture late doses to include a value for delayed MCV1 doses in the next field. The regional JRF for the Americas concerning delayed MCV1 and DTP3 does not include the logical skip pattern implemented in the global version in 2020.

**Figure 1.**
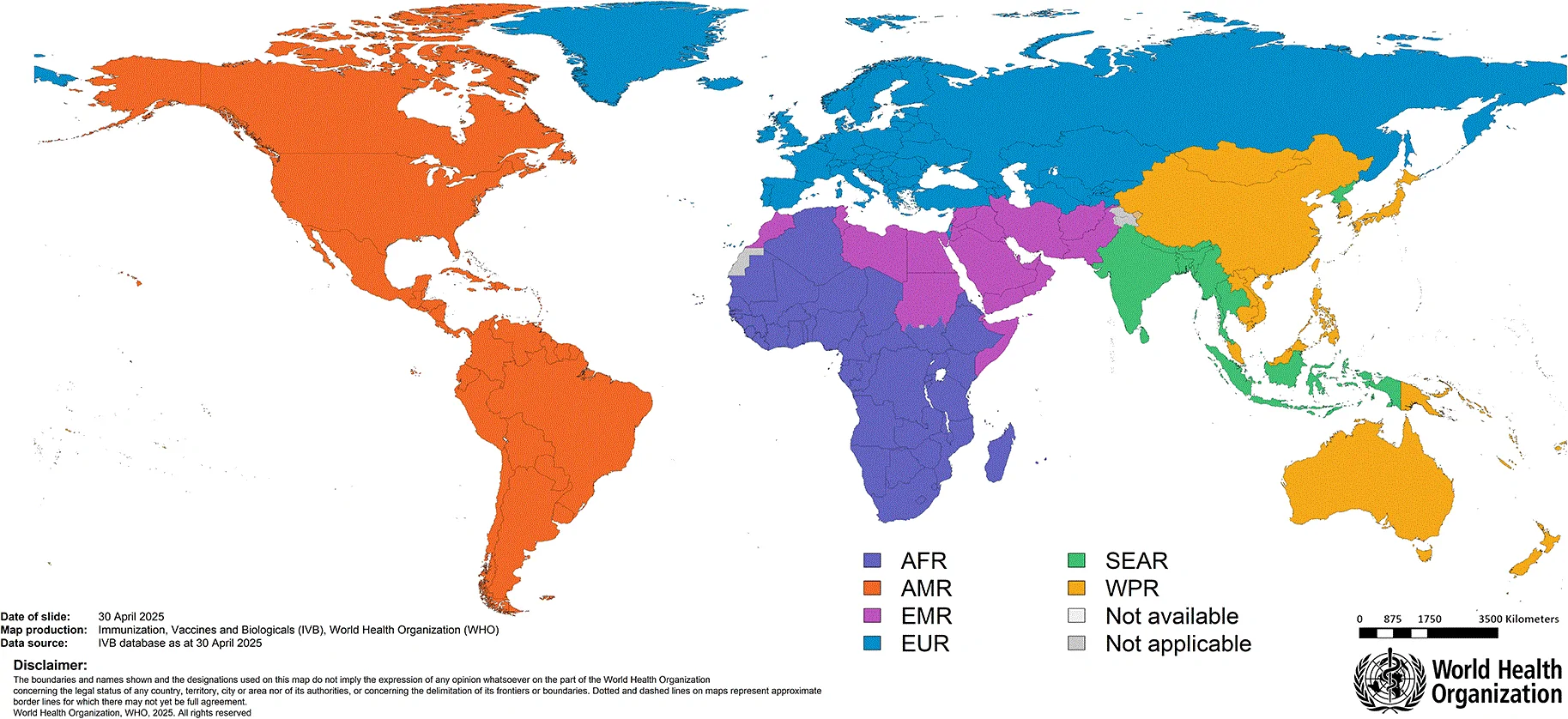
Regions of the World Health Organization, 2023.

We present proportions of countries reporting having systems to capture delayed doses by year, by WHO Region ( Figure 1), by World Bank Income Classification (2023 revision) and by Gavi-eligibility status in 2023. We also present the proportions of MCV1 doses that were “delayed” by dividing that number by the sum of “timely” plus “delayed” MCV1 doses.

Both timely and delayed doses were assigned to the cohorts according to the cohort year considering the vaccination schedule of each country. As an example, for a country that recommends MCV1 in infancy (i.e. 9-11 months), timely doses reported for 2020 were assigned to the 2020 birth cohort, and delayed doses administered between 12-23 months reported for 2020, were assigned to the 2019 birth cohort.

We explored whether population size affected the proportion of delayed doses by excluding countries with small populations (<10,000 as an arbitrary number and <16,862, as the last quartile of the distribution of population size of surviving infants), using the United Nations population estimates of surviving infants (2022 Revision).

Four additional indicators were estimated for countries reporting delayed MCV1 doses: (i) the percentage of doses administered according to time of administration (timely and delayed), (ii) coverage considering only timely doses, (iii) coverage considering timely and delayed doses, and (iv) difference between the two coverages (iii minus ii). The difference between the two coverages can be interpreted as the possibility for countries to improve their vaccination coverage with catch-up activities. We selected four countries with different recommended ages for MCV1 and with available data to illustrate in a single figure, this concept.

Global data we extracted in .csv from the WHO Immunization Information System data warehouse or WIISE Mart (on 25 Aug 2023) and PAHO data was extracted from the Regional JRF database (on 25 October 2023). Analyses and visualizations were done using RStudio statistical software (version 2023.06.1, Posit, PBC).

## Results

### Data completeness and quality

Of the 194 WHO Member States, 191 submitted the JRF for 2016, 194 for 2017 and 2018, 183 for 2019, 184 for 2020, 188 for 2021, and 186 for 2022.

All Member States completed the question on whether the information system allowed capturing data on late dose in the period 2016 to 2022 at least once; 126 countries (64.9%) had complete data for the entire period; 174 (89.7%) reported data for at least five of the seven years included. Of the countries that reported all years, 33 (26.2%) reported “yes” and subsequently “no”, which we interpret as inconsistent reporting, eight countries reported “no” and subsequently “yes” which we take to mean they had started implementing a recording system, with seven of those countries reporting delayed MCV1 doses for 2022.

**Figure 2.**
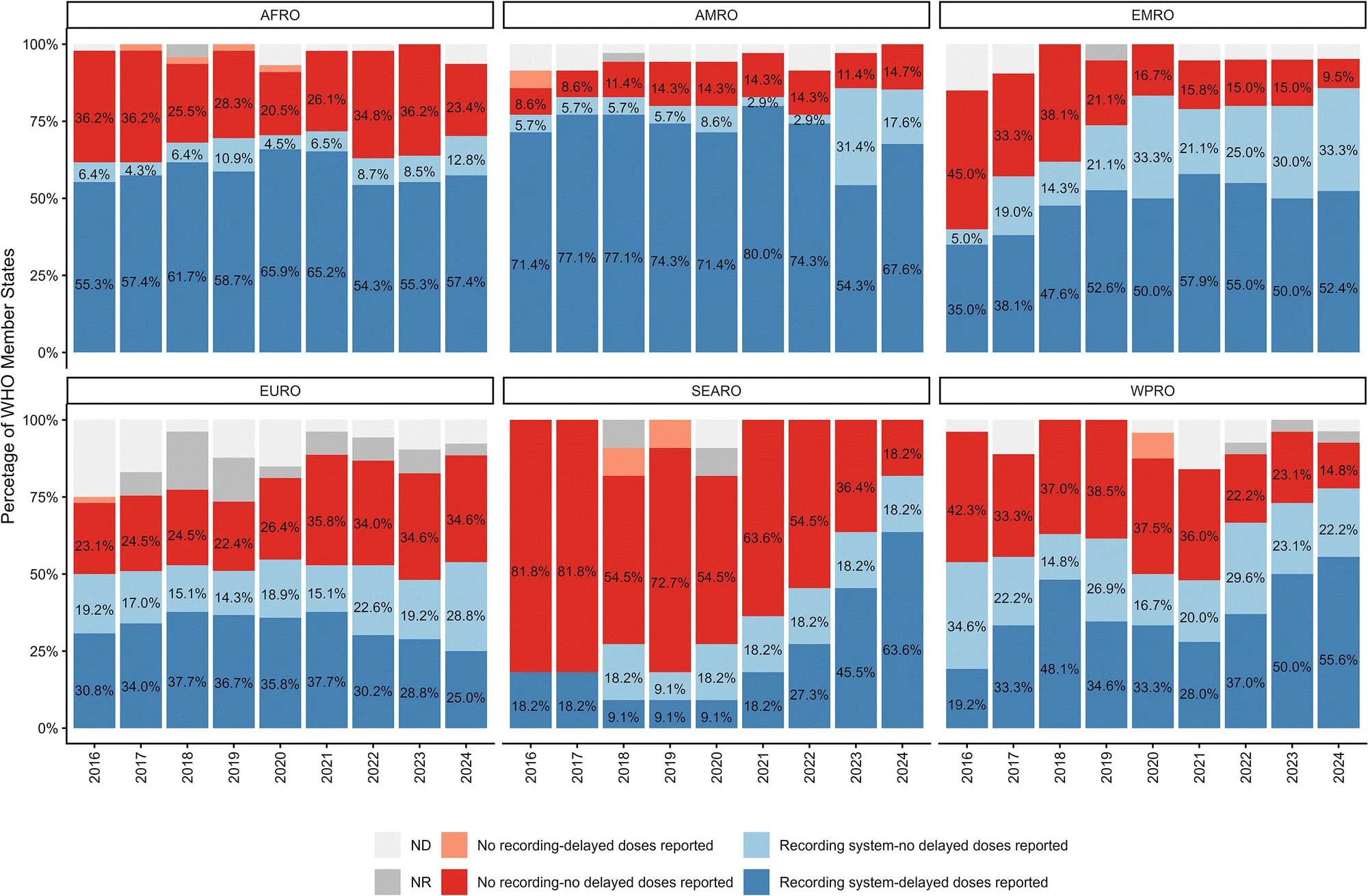
Responses to the question “ *Does your recording system allow for capture of data, at the national level, on the number of delayed or late doses administered?*”, Member States by WHO Region, 2016-2022.

The proportion of Member States reporting that their information system could capture delayed doses increased from 55.5% in 2016 to approximately 64% in 2022, though the proportions varied between years. Figure 2 shows the responses over time. Table 1 presents similar data at the global level and by country grouping highlighting variations in response rates by country category. The Region of the Americas (AMR) had the highest proportion of countries reporting having a system that allowed capturing data on the number of delayed doses administered, and the lowest proportion was in the South-East Asian Region (SEAR). About half of high-income countries (HICs) indicated having a system, while low-income countries increasingly reported the ability to capture delayed doses (up to 84% in 2021). The low proportion in HICs can, at least in part, be explained by the fact that 14 of 59 HICs do not use administrative information systems for immunization and rely primarily on surveys to monitor coverage. Median percentages of delayed doses were broadly similar between large and small countries, although small countries tended to show greater variability and somewhat higher values in recent years.

**Table 1.** Analysis of JRF data for Delayed MCV1 vaccination at global level and by country grouping, 2016-2022.

System allows for capturing data on the number of delayed doses administered			Year						
			2016	2017	2018	2019	2020	2021	2022
**Overall (N = 194)**		Number of countries reporting	191	194	194	183	184	188	186
		N (% of those reporting) countries responding “Yes”	106 (55.5)	114 (58.8)	122 (62.9)	117 (63.9)	118 (64.1)	121 (64.4)	119 (64.0)
		N countries reporting delayed doses (% of those reporting)	74 (38.7)	92 (47.4)	100 (51.5)	94 (51.4)	95 (51.6)	98 (52.1)	88 (47.3)
		Percentage of delayed doses reported, median (p25-p75) *	4.2 (1.6-9.8)	4.3 (1-8)	4.3 (1.7-13.2)	3.7 (0.9-9.7)	4 (0.9-17.2)	4 (1.4-13.9)	6.3 (1.9-19.6)
**WHO Region**	** AFR (N = 47)**	Number of countries reporting	47	47	47	46	43	46	46
		N (%) countries responding “Yes”	29 (61.7)	29 (61.7)	32 (68.1)	32 (69.6)	31 (72.1)	33 (71.7)	29 (63.0)
		N countries reporting delayed doses	23 (48.9)	27 (57.4)	28 (59.6)	27 (58.7)	29 (67.4)	30 (65.2)	24 (52.2)
		Percentage of delayed doses reported, median (p25-p75)	1.8 (0.9-7.8)	1.2 (0.7-4.4)	2.9 (1.1-5.9)	1.8 (0.9-5.8)	1.2 (0.5-2.7)	1.5 (0.7-3.5)	1.8 (0.7-3.8)
	**AMR (N = 35)**	Number of countries reporting	35	35	35	35	35	35	33
		N (%) countries responding “Yes”	27 (77.1)	29 (82.9)	29 (82.9)	28 (80.0)	28 (80.0)	29 (82.9)	26 (78.8)
		N countries reporting delayed doses	26(74.3)	31 (88.6)	32 (91.4)	29 (82.9)	28 (80.0)	31 (88.6)	29 (87.9)
		Percentage of delayed doses reported, median (p25-p75)	4.1 (2.1-8.8)	4.9 (2.2-6.8)	3.6 (1.8-8.6)	3.7 (1.9-9.5)	7.2 (3.6-19.8)	7.8 (2.9-23.6)	9.2 (6.1-20.7)
	**EMR (N = 21)**	Number of countries reporting	20	21	21	19	18	19	19
		N (%) countries responding “Yes”	8 (40.0)	12 (57.1)	13 (61.9)	14 (73.7)	15 (83.3)	15 (78.9)	15 (78.9)
		N countries reporting delayed doses	6 (30.0)	7 (33.3)	9 (42.9)	10 (52.6)	9 (50.0)	10 (52.6)	8 (42.1)
		Percentage of delayed doses reported, median (p25-p75)	4.1 (1.1-9.1)	5.2 (1-11.8)	4.6 (0.1-12.6)	1.4 (0.2-4.6)	3.7 (0.6-25.4)	2.4 (0.2-11)	7.5 (0.9-23.1)
	**EUR (N = 53)**	Number of countries reporting	52	53	53	46	53	53	50
		N (%) countries responding “Yes”	26 (50.0)	27 (50.9)	28 (52.8)	25 (54.3)	29 (54.7)	28 (52.8)	26 (52.0)
		N countries reporting delayed doses	13 (25)	16 (30.2)	18 (34)	18 (39.1)	19 (35.8)	19 (35.8)	15 (30.0)
		Percentage of delayed doses reported, median (p25-p75)	7.5 (4.6-22.1)	4.3 (0.6-11.6)	14.6 (5.4-28.4)	9.2 (1.6-19.4)	12.1 (1-27.5)	18.3 (2.8-26.8)	12.2 (2.1-24.6)
	**SEAR (N = 11)**	Number of countries reporting	11	11	11	11	11	11	11
		N (%) countries responding “Yes”	2 (18.2)	2 (18.2)	3 (27.3)	2 (18.2)	3 (27.3)	4 (36.4)	5 (45.5)
		N countries reporting delayed doses	1 (9.1)	1 (9.1)	1 (9.1)	1 (9.1)	0 (0.0)	1 (9.1)	2 (18.2)
		Percentage of delayed doses reported, median (p25-p75)	NC	NC	NC	NC	NC	NC	NC
**WPR (N = 27)**	Number of countries reporting	26	27	27	26	24	24	27
	N (%) countries responding “Yes”	14 (53.8)	15 (55.6)	17 (63.0)	16 (61.5)	12 (50.0)	12 (50.0)	18 (66.7)
	N countries reporting delayed doses	5 (19.2)	10 (37)	12 (44.4)	9 (34.6)	10 (41.7)	7 (29.2)	10 (37.0)
	Percentage of delayed doses reported, median (p25-p75)	4.2 (3.8-21.1)	20.2 (7.5-34.5)	9 (5-21)	7.9 (6.2-13.5)	12.8 (4.6-58.7)	5 (3.5-9.8)	17.6 (5.3-29.8)
**World Bank Income Classification** **^**	**Low (N = 26)**	Number of countries reporting	26	26	26	26	25	26	25
		N (%) countries responding “Yes”	16 (61.5)	17 (65.4)	16 (61.5)	16 (61.5)	21 (84.0)	22 (84.6)	19 (76.0)
		N countries reporting delayed doses	12 (46.2)	14 (53.8)	12 (46.2)	12 (46.2)	16 (64.0)	17 (65.4)	15 (60.0)
		Percentage of delayed doses reported, median (p25-p75)	2.7 (0.9-7)	1.8 (0.5-4.7)	3.2 (2.5-7)	1.5 (0.6-7.5)	0.9 (0.4-1.9)	1.4 (0.7-3.5)	3.6 (1.5-16.2)
	**Lower-middle (N = 54)**	Number of countries reporting	54	54	54	53	50	51	52
		N (%) countries responding “Yes”	29 (53.7)	32 (59.3)	36 (66.7)	37 (69.8)	30 (60.0)	30 (58.8)	34 (65.4)
		N countries reporting delayed doses	20 (37)	29 (53.7)	31 (57.4)	27 (51)	26 (52)	24 (47.1)	26 (50.0)
		Percentage of delayed doses reported, median (p25-p75)	2.5 (0.9-11.1)	4.4 (0.9-13.8)	3.8 (1.1-12.6)	2.7 (1.7-8)	3.8 (1.5-23.9)	3.7 (1-9.5)	4.5 (1.6-15)
	**Upper-middle (N = 52)**	Number of countries reporting	52	52	52	49	51	51	52
		N (%) countries responding “Yes”	33 (63.5)	36 (69.2)	38 (73.1)	34 (69.4)	34 (66.7)	36 (70.6)	37 (71.2)
		N countries reporting delayed doses	29 (55.8)	33 (63.5)	33 (63.5)	31 (63.3)	30 (58.8)	32 (62.7)	27 (51.9)
		Percentage of delayed doses reported, median (p25-p75)	4.3 (2.3-8.1)	4.7 (1.5-8)	4.2 (1.3-18)	3.7 (0.7-10.3)	5.9 (1.1-23.6)	8.3 (2.9-26.7)	9.7 (4.3-32.5)
	**High (N = 59)**	Number of countries reporting	56	59	59	52	57	58	55
		N (%) countries responding “Yes”	26 (46.4)	26 (44.1)	29 (49.2)	27 (51.9)	32 (56.1)	31 (53.4)	27 (49.1)
		N countries reporting delayed doses	12 (21.4)	14 (23.7)	20 (34)	20 (38.5)	21 (36.8)	21 (36.2)	18 (32.7)
		Percentage of delayed doses reported, median (p25-p75)	5.4 (3.9-13.2)	5 (3-6.1)	5 (2.1-11)	5.5 (2.7-10.2)	6.9 (2.8-14.5)	4.1 (1.7-14.2)	6.3 (2.5-7.5)
**Gavi-eligibility **	**Yes (N = 57)**	Number of countries reporting	57	57	57	57	55	57	55
		N (%) countries responding “Yes”	34 (59.6)	35 (61.4)	37 (64.9)	37 (64.9)	37 (67.3)	39 (68.4)	37 (67.3)
		N countries reporting delayed doses	25 (43.9)	30 (52.6)	31 (54.4)	27 (47.4)	30 (54.5)	31 (54.4)	29 (52.7)
		Percentage of delayed doses reported, median (p25-p75)	1.8 (0.8-6.8)	2.1 (0.7-4.9)	2.9 (1.1-7.1)	1.9 (0.9-6)	1.3 (0.7-6.1)	1.9 (0.8-5.8)	2.7 (1.1-9.6)
	**No (N = 137)**	Number of countries reporting	134	137	137	126	129	131	131
		N (%) countries responding “Yes”	72 (53.7)	79 (57.7)	85 (62.0)	80 (63.5)	81 (62.8)	82 (62.6)	82 (62.6)
		N countries reporting delayed doses	49 (36.6)	58 (42.3)	64 (46.7)	63 (50)	61 (47.3)	62 (47.3)	54 (41.2)
		Percentage of delayed doses reported, median (p25-p75)	4.9 (2.3-16.3)	5.2 (1.5-11)	5.5 (1.8-18.4)	4.8 (0.8-12.8)	6.6 (2.1-23.7)	7.1 (1.7-25.1)	9.5 (3.2-25.8)

			2016	2017	2018	2019	2020	2021	2022
**Population size**	Large	N countries delayed doses reported	63	72	73	71	74	75	70
		p50 (p25-p75) percentage of delayed doses reported	4.3 (1.5-12.3)	4.2 (0.9-8)	4.2 (1.1-12.6)	4 (1-11.2)	3.6 (0.8-19.1)	3.9 (1.4-12.9)	5.5 (1.7-18.5)
	Small	N countries delayed doses reported	11	18	21	21	18	19	18
		p50 (p25-p75) percentage of delayed doses reported	3.4 (2.3-6)	4.9 (2.2-24.6)	4.4 (2.8-18)	5.2 (0.9-11.6)	7.6 (4-15.3)	5 (1.5-9)	9.7 (3.7-21.3)

AFR: African Region; AMR: Americas Region; EUR: European Region; WPR: Western Pacific; SEAR: South-East Asian Region; EMR: Eastern Mediterranean Region.

NC: Not calculated due to small N.

* Percentile 25-percentile75.

^**^**^ Excludes three WHO member states that were not WB classified in 2023.

**Figure 3.**
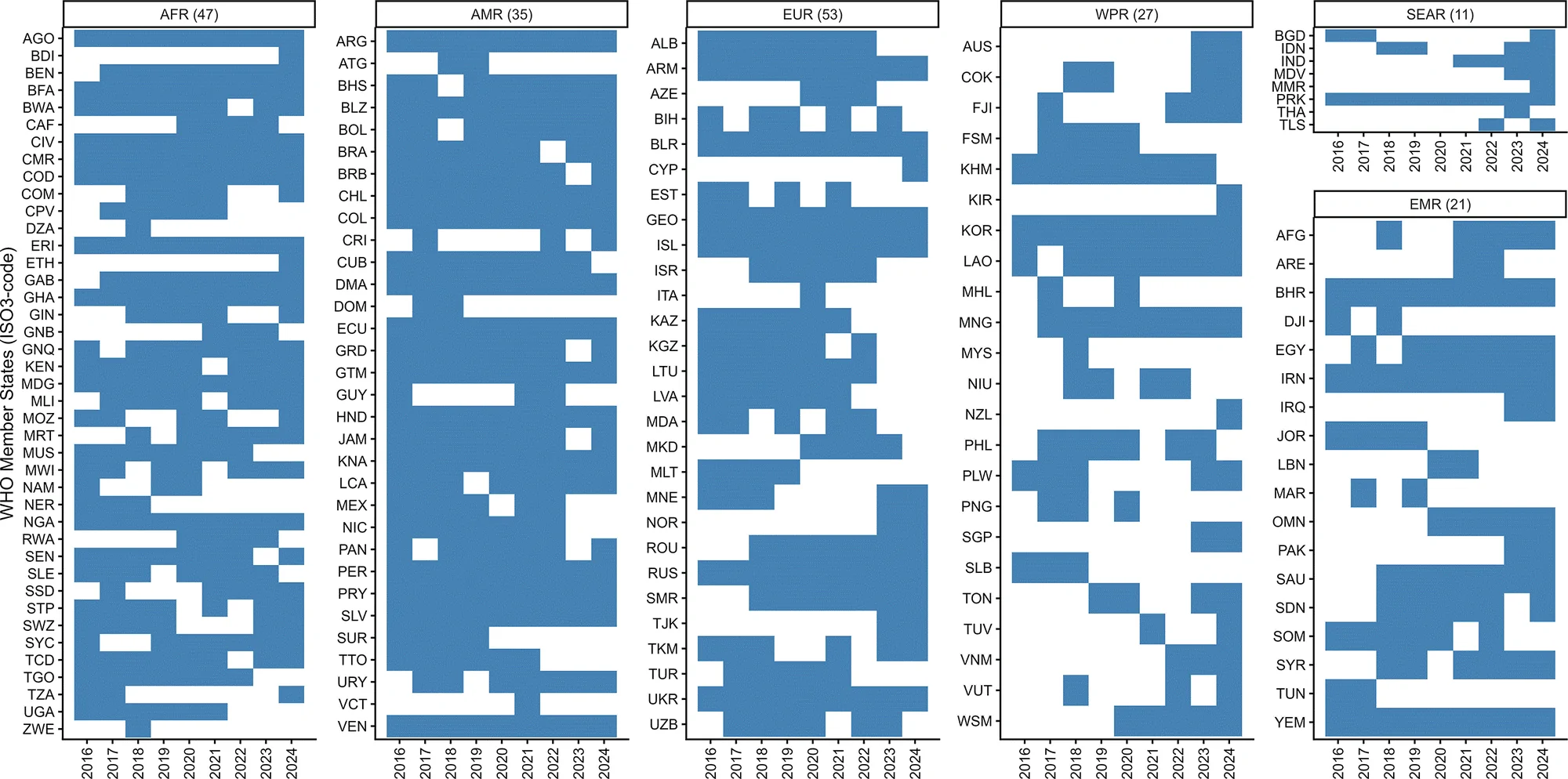
WHO Member States reporting delayed MCV1 doses, by WHO Region, 2016-2022. WHO Regions are ordered alphabetically except for SEAR and EMR that are displayed last and in the same column to optimize the size and shape of the figure. AFR: African Region; AMR: Americas Region; EUR: European Region; WPR: Western Pacific; SEAR: South-East Asian Region; EMR: Eastern Mediterranean Region.

Of the 194 WHO Member States, 136 (70.1%) reported at least once the number of delayed MCV1 doses administered. This proportion of reporting varied importantly by WHO Region and by year ( Figure 3). A mismatch between the response regarding having a system to capture late doses and then providing the number of late doses was seen in 189 instances (of 1,320 JRFs for the period 2016-2022), mainly reflecting countries that reported “yes” to having a system to capture delayed doses, and then not providing a data on delayed MCV1 doses, but with 11 of those instances being a “no” to having a system to report late doses and then adding data on delayed MCV1 doses. We did not identify any clear pattern in the types of countries with inconsistent reporting (the GitHub repository includes tables with this data).

In 52 instances, the value reported for delayed doses was zero, and in 119 instances, no data was provided in the JRF for timely MCV1 doses. This lack of data impeded the calculation of the proportion of late doses relative to the total MCV1 doses. Excluding these instances, the median proportion of delayed MCV1 vaccinations was 4.4% (interquartile range of 1.2% to 13.8%) ( Table 2). There were 46 instances where the percentage of late doses was 50% or higher, likely due to errors reflecting the same number of doses reported as both “on time” and “delayed.” When excluding these 46 instances, the median 3.9% (interquartile range 1.1% - 9.5%).

**Table 2.** Differences in MCV1 coverage considering delayed doses reported by birth cohort overall and by country groupings for countries reporting data on timely and delayed doses, 2017-2021.

			Birth cohort year				
			2017	2028	2019	2020	2021
Overall		N of countries *	58	74	85	77	25
		Coverage difference, median (p25-p75) **	5.1 (1.8-11.5)	4.2 (1.1-13.7)	5 (1.5-27.4)	4.8 (1.3-11)	3.6 (0.6-21.5)
WHO Region	AFR	N of countries	NA	17	19	16	15
		Coverage difference, median (p25-p75)	NA	2.1 (0.6-3.4)	1.5 (0.6-3.5)	0.5 (0.3-2.6)	1.1 (0.4-2.9)
	AMR	N of countries	30	25	29	30	2
		Coverage difference, median (p25-p75)	3.6 (1.8-7.9)	5.3 (1.5-10.8)	9.9 (2-42.3)	5.5 (2.7-11)	NA
	EMR	N of countries	5	6	8	7	2
		Coverage difference, median (p25-p75)	0.7 (0.5-3.7)	3.1 (1-5.1)	2.4 (0.5-23.3)	6.9 (0.8-25.4)	NA
	EUR	N of countries	19	16	19	17	1
		Coverage difference, median (p25-p75)	8.2 (3.5-20.1)	14.6 (2.9-24.1)	8.1 (2.5-29)	12.8 (2.5-34.4)	NA
	SEAR	N of countries	NA	1	0	0	1
		Coverage difference, median (p25-p75)	NA	NA	NA	NA	NA
	WPR	N of countries	4	9	10	7	4
		Coverage difference, median (p25-p75)	21.9 (7.2-86.6)	8 (4.9-16.2)	11.4 (3.7-49.1)	5 (3.9-7.8)	16.6 (10-39.4)
World Bank Income Classification **^**	Low	N of countries	NA	6	10	8	6
		Coverage difference, median (p25-p75)	NA	1.9 (0.3-5.1)	1 (0.3-3.3)	1 (0.3-3.3)	1.7 (0.7-6.5)
	Lower-middle	N of countries	9	22	21	21	12
		Coverage difference, median (p25-p75)	3.8 (2.2-36)	6 (2.2-13.3)	5 (1.5-66.2)	3.4 (0.9-8.2)	4.2 (1.1-9.8)
Upper-middle	N of countries	26	25	32	28	4
	Coverage difference, median (p25-p75)	4.2 (0.9-11)	2.5 (0.5-6.8)	8.5 (2.8-35.7)	7.9 (3.9-21.8)	82 (56.8-90)
High	N of countries	19	17	20	18	2
	Coverage difference, median (p25-p75)	4.8 (2.5-8.5)	6.9 (2.7-27.7)	3.4 (1.4-11.2)	3.3 (1.6-6.9)	NA
Gavi-eligibility	Yes	N of countries	3	17	21	18	14
		Coverage difference, median (p25-p75)	NC	3 (1-9.4)	3.2 (0.8-11.8)	1.6 (0.3-6.4)	1.4 (0.6-5.2)
	No	N of countries	51	53	61	53	11
		Coverage difference, median (p25-p75)	4.5 (1.4-11.4)	4.7 (0.9-16.2)	6.1 (2-30.1)	5.5 (2.4-21.1)	11.7 (2.7-82.1)

AFR: African Region; AMR: Americas Region; EUR: European Region; WPR: Western Pacific; SEAR: South-East Asian Region; EMR: Eastern Mediterranean Region.

NA: Not available;

NC: Not calculated due to small N.

* N of countries refers to countries reporting timely and delayed MCV1 doses.

** Percentile 25-percentile75.

^ Excludes three WHO member states that were not WB classified in 2023.

The annual number of instances where vaccination coverage by birth cohort could be calculated accounting for both timely and delayed doses was low, varying between 25 and 85. Table 2 presents the median and interquartile range of the difference between coverage calculated using only on-time doses and coverage calculated using both on-time and delayed doses. The overall median differences fluctuated between 3.6% and 5.1% across the years available, indicating that if countries continued monitoring coverage as the cohort ages coverage would increase by not an insignificant proportion. In all cohort years, the median coverage increases when including delayed or catch-up doses, was smaller in Gavi-eligible countries compared to non-Gavi-eligible countries. Proportion of delayed MCV1 vaccination did not change removing countries with small cohort size (data not shown).

**Figure 4.**
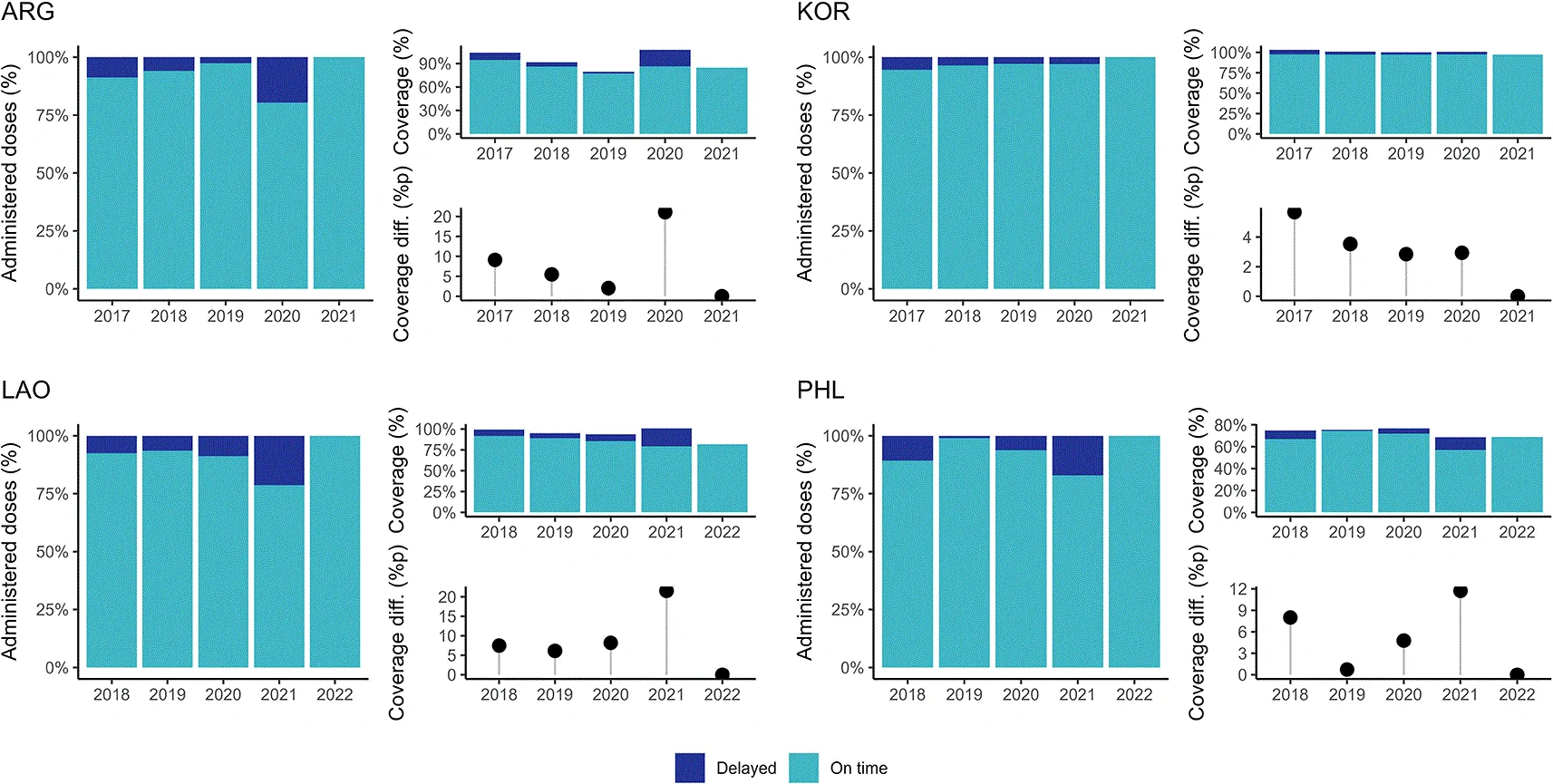
First measles-containing vaccine (MCV1) doses administered and coverage, on-time and delayed, by birth cohort in selected countries (ARG, KOR, LAO, PHL), birth cohorts 2017-2021 for countries recommending MCV1 in the second year of life and 2018-2022 for those recommending MCV1 in infancy. The last year is included only to illustrate coverage in the second graph, as delayed doses for that cohort were not available yet at the time of data extraction.

Figure 4 includes bar charts and scatter plots for four countries (for illustration purposes): Argentina (ARG), South Korea (KOR), Lao People’s Democratic Republic (LAO), and the Philippines (PHL); with the first two countries recommending MCV1 in the second year of life, and the other two in infancy. Each country has three graphs, all with the x-axis reflecting the birth cohort of the children receiving the doses. ARG and KOR start with the 2017 cohort and LAO and PHL with the 2018 cohort. The bars show the “delayed” doses in blue and “on time” doses in teal, the first graph depicts the proportion of all MCV1 doses given and the next one, as the coverage reached for the cohort. The scatter plots show MCV1 coverage difference (%) when monitoring only for one year vs. what the coverage is for the cohort when catch-up doses are added. The last cohort included in the graphs does not yet have the data to add the catch-up doses. For ARG, LAO and PHL, the figures suggest that the 2020 cohort had a higher proportion of children who were caught-up, with ARG and LAO able to increase by close to 20%. Whereas in KOR, there are fewer fluctuations.

Of the 35 WHO Member States of the Americas, all recommend DTP3 in infancy and MCV1 in the second year of life, with the exception of Haiti that recommends MCV1 at 9 months of age. Canada and the United States use surveys for vaccination coverage monitoring and thus do not report on number of delayed doses. Of the 33 countries in Latin America and the Caribbean (LAC), only Haiti has reported not having a system that allows monitoring late doses, yet delayed doses were reported for 2009 and 2014. All LAC countries reported at least one year of delayed doses for MCV1 and 30 reported delayed DTP3 doses at least once for the period of 2006-2022. No country reported their MCV1 delayed doses for the entire period 2006-2022 but 14 countries reported such doses for at least 10 of those years (for 4 countries those values were zero). This was similar for delayed DTP3 doses, where 15 countries reported on delayed doses for at least years.

**Figure 5.**
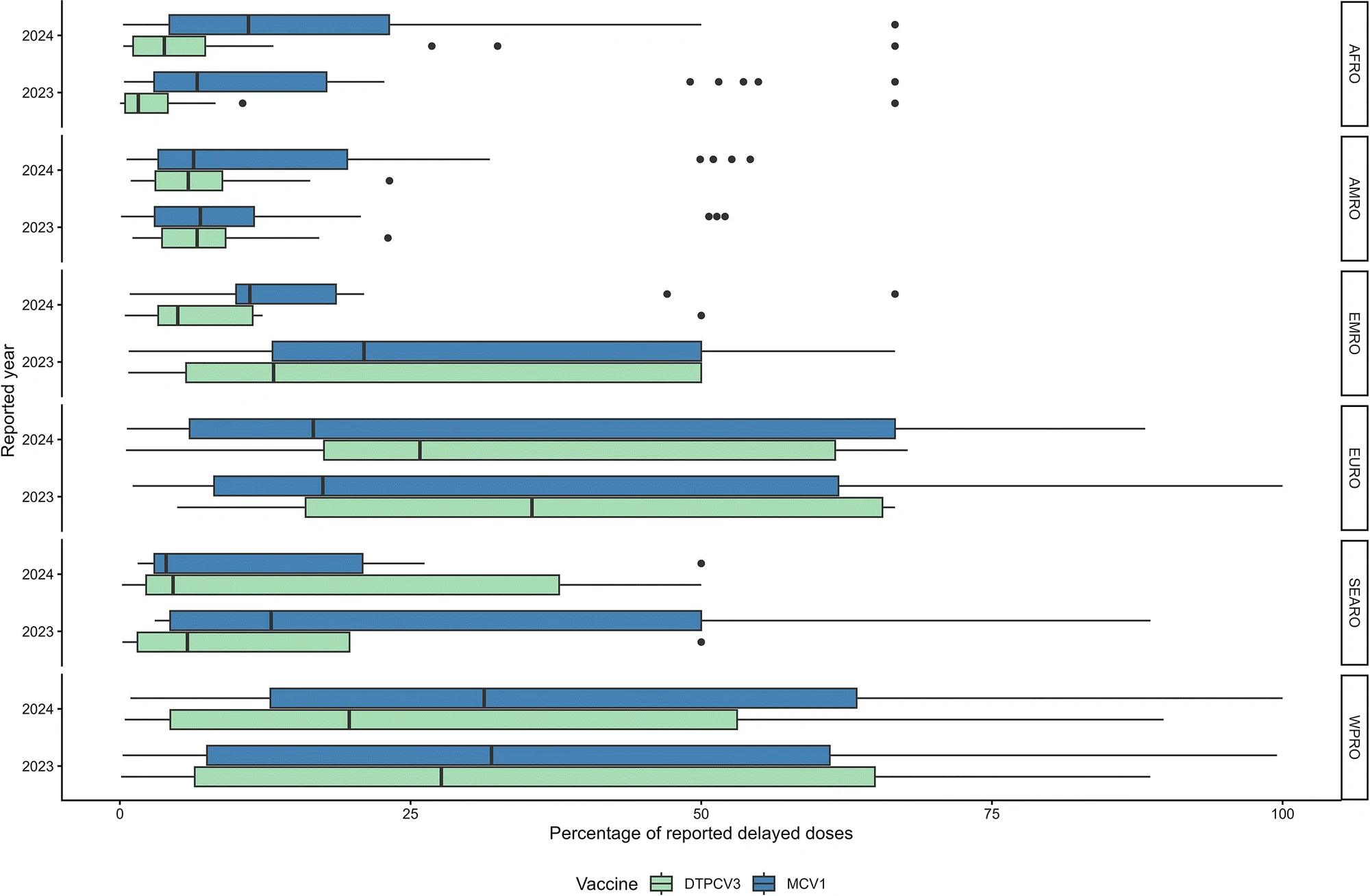
Median, interquartile distribution and range of the percentage of reported delayed third doses of diphtheria-tetanus-pertussis vaccines (DTP3) and first measles-containing vaccines (MCV1), Latin American and Caribbean countries, 2006-2022.

When comparing DTP3 and MCV1 reporting in LAC throughout the reporting years, the completeness and magnitude of reporting of delayed doses of DTP3 fluctuate less than for MCV1 doses suggesting timelier DTP3 vaccination than MCV1 vaccination across LAC countries ( Figure 5).

## Discussion

This is the first global analysis of countries’ reported ability to capture late childhood vaccination doses and the to explore the percentage of delayed, or more positively “catch-up”, MCV1 doses based on administrative data. It is also the first to quantify the reported proportion of delayed MCV1 and DTP3 doses in Latin America and the Caribbean. Even with some incomplete reporting, data suggest that in 2022, over two thirds of all WHO Member States had an information system able to capture delayed MCV1 doses. However, half or less then go on to report a number for late doses, and of those, the proportion of total doses given late varies widely. Furthermore, there are important inconsistencies and completeness issues that raise concerns about data quality that will need to be further explored to understand why reports of delayed doses seem to have data quality issues.

Timely vaccination is important, as it reduces the time that children spend unnecessarily vulnerable to vaccine-preventable diseases (VPDs). ^23, 24^ Data from surveys demonstrate variability in the proportion of children who are vaccinated after the first year of life, ^22, 25, 26^ with children in rural areas and those from deprived socioeconomic backgrounds having more protracted delays. ^25, 27– 30^ Delayed vaccination has been associated with not completing vaccination schedules, even in developed countries. ^31, 32^ Nevertheless, receiving a vaccine late is better than not receiving it, ^18^ and catch-up vaccination has been at the forefront of the global immunization discourse following the declines in coverage seen during the Covid-19 pandemic and the ensuing VPD outbreaks. ^15– 19, 33– 36^

While our summary suggests that many countries had a system allowing separate recording of MCV1 doses for those aged <12 months and those aged >=12 months (or 12-23 m and more in cases where MCV1 is recommended in the second year of life). The small percentage may be taken to reflect at face value low catch-up rates, or it may result from under-reporting or misclassification of delayed doses as being timely. Only few analyses have compared the reported proportion of delayed doses using administrative data, i.e., data on vaccine doses given, collected and recorded at point of service, and the proportion of late vaccination seen in surveys. In such analyses, under-reporting of late vaccination has been observed. ^37, 38^ Until the 2023 Catch-up efforts, not much attention had been given to the quality of delayed/catch-up vaccination data at the global level. The underlying reasons for the inconsistencies observed in the reported, and the differences seen in admin data vs. surveys, are being explored, for example through trying to better understand the design of tally sheets ^39^ and through documenting the challenges in data systems seen in countries that Gavi supported in the context of the “Big Catch-up”. ^40^

As other literature suggests and our analysis illustrates, the current practice of monitoring coverage for one calendar year may underestimate the final coverage reached for a given birth cohort and may limit understanding the magnitude of delayed vaccination. For example, if children born in 2020 had been missed that year, but caught-up in 2021, only referring to single-year coverage estimates per calendar year will miss to account for children vaccinated after their first birthday, thus potentially underestimating the real proportion of the cohort vaccinated by a later age (e.g., by age 59 months). While monitoring how a cohort continues to be vaccinated is feasible using survey data (for children with documented dates of birth and dates of vaccination) and electronic immunization registries, ^23, 41^ the current aggregated information systems limit the ability of immunization program managers to monitor the occurrence of delayed vaccination and the effect of catch-up activities in the protection of the population. Furthermore, issues with aggregated immunization information systems are those of health management information systems, such as poor data quality, issues with interoperability, human resource constraints, and high financial costs that affect maintenance and, when reliant on donor support, sustainability. The WHO SCORE initiative provides a useful framework and evaluation of countries capacities to produce, manage and use health data for decision-making. The 2025 SCORE report highlighted the important differences seen between high and low-income countries. ^42^ As most immunization programs finish transitioning from a focus on childhood to a life course approach, and vaccines targeting broader age groups are introduced, the more important it becomes to be able to transition to a cohort approach for monitoring vaccination, ^43^ and the use of electronic immunization registries, where feasible and appropriate. ^44, 45^ This has been illustrated by HPV coverage estimation, that includes monitoring coverage by age 15 years. ^46^

Following this analysis, and in the context of the Big Catch-up initiative, WHO and UNICEF modified the relevant questions in the eJRF. The 2024 version of the eJRF, for 2023 data, included the following questions: 1) “ *Does your recording system allow for capture of data, at the national level, on the number of delayed or late doses administered?”* (instructions and definitions were added); 2) [If “not” in the previous question] *Are there plans to capture data on the number of delayed or late doses administered in your recording system in the future?*; 3) [If “yes” in the previous question] *“What year do you plan on implementing a system to capture delayed or late doses?”*; 4) How many doses of DTP3 were administered in {year} at: 12-23 m and >=24 months (or >=12 months were the previous disaggregation is not available) (instructions are also included). The questions on delayed MCV1 were maintained though the age groups were aligned to those of DTP3 and instructions were added. Furthermore, the JRF now also requests a copy of the tally sheets, or equivalent primary data collection tools, to provide a better overview of tally sheet design and age disaggregation. This has helped inform some of the changes to data collection tools and health management information systems needed to implement catch-up activities, particularly in countries receiving support from Gavi, the Vaccine Alliance for Big Catch-up implementation. It is expected that a repository of these data collection tools be created, similar to what exists for vaccination cards or home-based records. ^47^

Even though the response rate to the two relevant questions of the JRF was quite high, the findings of this analysis are subject to important limitations. First, completeness is not universal. Second, this analysis used reported data and errors in interpretation by country officials completing the JRF may have occurred. Third, almost 1 in 4 countries were flagged as having inconsistent responses to the system question that jumped from “yes” to “no”, which is very unlikely based on how information systems are rarely changed to remove age-groups. This likely highlights issues with the explanations included in the JRF about the requested information. Fourth, for several years the questionnaire allowed reporting a number of delayed doses even if the question on having a system was “no” or left unanswered or reported as not relevant (NR). Fifth, low proportions of delayed doses likely reflect incomplete or erroneous reporting and high proportions are likely a reflection of errors in understanding the data being requested. Sixth, there has not been any systematical approach to explored delayed/catch-up vaccination among vulnerable groups implications for vulnerable groups (children in conflict zones, migrant communities) and one may speculate that they may be more likely to miss timely doses. Finally, to our knowledge, no country has undertaken systematic validations on accuracy of recording late doses on the tally sheets at the point of vaccination; immunization data quality assessments have historically focused solely on timely doses. ^48^ We do believe that our analysis illustrates what is reported as serves a starting point to advance the discussion and to serve as a call to strengthen immunization data systems and to monitor vaccination coverage beyond infancy.

## Conclusion

Most countries have a system allowing the capture of doses administered late, yet not all. Also, the low proportion of countries reporting on delayed doses administered each year likely reflects barriers to aggregating data by age-group and a historical lack of attention given to catch-up or late vaccination. As countries continue to strengthen routine immunization programs, including catch-up activities, and move towards a life-course approach for immunization, adapting immunization information systems will be needed to improve the monitoring of these activities.

## Ethics and consent

No individual-level data were used in this study. Only country-level data officially reported by Ministries of Health to WHO and UNICEF were used. As a result, ethical approval and consent was not required.

## Reporting guidelines

Not available.

## Disclaimer

The authors alone are responsible for the views expressed in this article and they do not necessarily represent the views, decisions or policies of the institutions with which they are affiliated.

## Data Availability

All data used are publicly available at https://immunizationdata.who.int/. We also have organized all files and R code in GitHub for Open access here https://github.com/thiscarolina/Data_Delayed-Vaccination-Paper https://doi.org/10.5281/zenodo.15797724
